# Does cortisol mediate the association between school bullying and weight status in preadolescents?

**DOI:** 10.1007/s00787-026-02999-4

**Published:** 2026-04-08

**Authors:** Izaro Babarro, Ainara Andiarena, Jesus Ibarluzea, Maialen Otamendi, Mònica Guxens, Martine Vrijheid, Nerea Lertxundi

**Affiliations:** 1 Faculty of Medicine and Nursing, University of the Basque Country, UPV/EHU, 20014 Donostia/San Sebastian, Spain; 2 Group of Environmental Epidemiology and Child Development, Biogipuzkoa Health Research Institute, Donostia/San Sebastian, 20014 Spain; 3 Faculty of Psychology, University of the Basque Country, UPV/EHU, Donostia/San Sebastian, 20018 Spain; 4 Biomedical Research Centre Network for Epidemiology and Public Health (CIBERESP), Madrid, 28029 Spain; 5 ISGlobal, Barcelona, 08003 Spain; 6 Universitat Pompeu Fabra (UPF), Barcelona, 08003 Spain; 7 Department of Child and Adolescent Psychiatry/Psychology, Erasmus MC University Medical Centre, Roterdam, 3015GE The Netherlands; 8 ICREA, Barcelona, Spain

**Keywords:** Cortisol, Bullying, Fat percentage, BMI, Preadolescents

## Abstract

Childhood obesity and bullying are interconnected public health issues that negatively influence both, physical and mental health. Stress could be an explanatory mechanism mediating the relationship between bullying victimization and obesity. Thus, this study aims to investigate whether hair cortisol levels mediate the association between bullying and weight status. Participants were 11-year-old preadolescents from the INMA project in Spain (*N* = 465). Bullying was assessed using the Olweus Bully Victim Questionnaire (OBVQ), while weight status was determined based on Body Mass Index (BMI) and body fat percentage. To study the complex associations path analysis was carried out. In terms of bullying prevalence, 8.6% of the participants were involved as victims, 1.7% as bullies and 2.4% as bully/victims. Regarding weight status, 24.9% of the participants were overweight and 11.4% had obesity. Results partially confirmed the main hypothesis, as a mediating effect of hair cortisol between involvement in bullying and body fat percentage was identified in boys, specifically those who were victims (*X*^2^ (1) = 0.326; *p* = 0.982; CFI = 1.00; RMSEA = 0.000; *p* = 0.987). Understanding this association is crucial for elucidating the mechanisms that may exist between bullying and some physical consequences of this phenomenon such as overweight or obesity.

## Introduction

 Bullying is defined as recurring behavior in school settings that involves intentional actions and an imbalance of power between the individuals involved [1]. Bullying is a complex social behavior that requires the integration of multiple theoretical perspectives to be fully understood [2]. One widely used framework is Bronfenbrenner’s ecological systems theory, which posits that human behavior cannot be fully understood without considering the influence of multiple layers of the environment. Applied to bullying, this framework highlights that such behavior is not an isolated individual phenomenon, but a complex process influenced by peer relationships, school climate, family factors, and broader societal norms [3]. Complementing this ecological perspective, individual factors—including biological ones—also play a key role. Research indicates that exposure to bullying is associated with alterations in physiological systems involved in stress regulation, including neuroendocrine and inflammatory processes [4, 5]. Within this integrative approach, Social Safety Theory proposes that humans are designed to seek and maintain safe and supportive social relationships. When these relationships are threatened by exclusion, rejection, or conflict, social stress is activated, triggering physiological responses [6] and when the stress is chronic can adversely affect both physical and mental health [7].

In line with this perspective, the association between bullying and cortisol levels has been explored in various studies, with two recent reviews concluding that there are mixed results [8, 9]. The inconsistencies in the associations may derive, among others, from the way cortisol levels were measured.

Studies using hair samples, suggest that although cumulative victimization was related to higher hair cortisol levels, peer victimization was not [10, 11]. Moreover, two studies reported that the association between victimization and hair cortisol concentrations was sex-dependent being only statistically significant for boys, with higher levels of victimization being associated with higher hair cortisol concentrations [10, 12]. In addition, in a previous study examining not only victimization but also other bullying roles, a trend association was observed between involvement as a bully/victim and higher hair cortisol concentrations [13]. Regarding cortisol assessed in saliva samples, studies focusing on cortisol reactivity following a stressful task generally found that victims exhibited lower cortisol reactivity compared to their non-victimized peers [14–19]. Other research has explored total cortisol levels, with most studies reporting that peer rejection or bullying involvement was associated with higher overall cortisol levels [20–22]. Finally, evidence from studies examining diurnal cortisol patterns indicates that bullied students tend to show flatter daily cortisol slopes [17, 21–23].

Obesity and bullying are two major public health issues during childhood and adolescence, given their high prevalence and significant consequences [24, 25]. These vulnerable developmental periods are characterized by biological, psychological, and social changes that can trigger variations in weight [26, 27]. Previous literature has attempted to explore how adverse experiences such as maltreatment or victimization get under the skin and affect health outcomes, including obesity [28, 29]. Miller and Lumeng in their study concluded that early life stress is associated with multiple biological and behavioral pathways in children that increase risk for later obesity, highlighting dysregulation of the hypothalamic-pituitary-adrenal (HPA) axis and cortisol secretion as a key mechanism [30]. In the same line cortisol has been shown to redistribute white adipose tissue to the abdominal area and increase appetite [31], and factors like a high glycemic diet, insufficient sleep, and high stress levels can increase this cortisol production [32].

Consistent with this, recent studies have demonstrated significant correlations between chronic cortisol levels and obesity in both adults [33] and children [34]. Consequently, several studies have investigated the mediating role of stress and cortisol in the relationship between maltreatment or victimization and weight status (BMI, overweight or obesity) [26, 35–38]. Although some profiles of the Hypothalamic-Pituitary-Adrenal (HPA) axis are believed to affect the risk of obesity following stressors such as maltreatment, the direction of the association remains unclear [39]. Few studies have directly explored the pathway linking childhood and adolescence victimization or maltreatment to obesity through cortisol or stress.

One study examined the potential mediating role of cortisol between childhood polivictimization and body mass index (BMI) in adulthood, specifically measuring the area under the curve (AUC) measured in saliva, finding that it was not statistically significant [40]. In contrast, a cross-sectional study conducted in children aged approximately 10 years discovered that cortisol, specifically Cortisol Awakening Response (CAR) mediated the relationship between childhood adversity and BMI [41].

Other studies exploring hair cortisol levels as a mediator between childhood maltreatment and BMI revealed that chronic cortisol did not mediate this association [37]. A sex specific association was observed in a study involving children around the age of 11 years. Specifically, in girls, cortisol reactivity moderated the association between maltreatment type and changes in BMI over time. At low levels of cortisol reactivity, girls who experienced sexual abuse showed a steeper quadratic increase in BMI compared with non-abused peers [36]. Finally, one study assessed the mediating role of perceived stress in this association between maltreatment and BMI and found that it also did not act as a mediator [38].

Regarding the association between bullying and weight status, although previous studies have demonstrated the association between being bullied during childhood and having overweight or obesity in the adulthood [42, 43]. To the best of our knowledge, a single study has previously examined the moderating effect of stress in this association, considering not only bullying but also the school environment as a potential source of stress [26]. This study suggests that stress related to school climate could influence weight status, only in girls. However, there is no previous research that has investigated the mediating role of cortisol on the relationship between the school contexts in general and bullying in particular with BMI.

Taken together, existing evidence suggests that bullying represents a chronic social stressor that may become biologically embedded through dysregulation of stress-related systems, particularly the HPA axis, in a manner that is complex and potentially sex-dependent. Given the established role of cortisol in metabolic regulation and weight-related outcomes, examining cortisol as a mediating mechanism linking bullying experiences to weight status is theoretically warranted.

A possible explanation for the discrepancies existing in the studied moderating association could be related to gender and sexual differences that occur during pubertal development. Firstly, hormonal fluctuations during puberty, impact appetite regulation and metabolism, intensifying the connection between stress and the risk of obesity [44]. Additionally, gender differences have been also identified in the way stress is perceived and managed. Females, particularly during adolescence, tend to be more sensitive to the emotional effect of stress [45], which may lead to unhealthy eating patterns such as preference for energy and nutrient dense foods [46]. In addition, children and adolescents’ health habits may be influenced by their primary caregivers, mainly mothers. Families with higher incomes tend to adopt healthier behaviors, such as making more appropriate dietary choices, engaging in more physical activity and better access to health services, all which contribute to reducing child BMI and reducing the risk of obesity [47, 48]. This underscores the importance of considering these factors as key variables in studies examining stress-related obesity.

Therefore, seeing the discrepancy that exists in the results and that there are no studies exploring the mediating role of cortisol in the relationship between bullying and BMI, our aim was to analyze the mediating role that cortisol plays in the relationship between bullying and weight status (BMI and percentage of body fat). In addition, the models were stratified by sex and we also considered pubertal status and maternal study level in the model.

## Methods

### Participants

The participants in the study were individuals from the cohorts of Gipuzkoa (Basque Country, Spain) and Sabadell (Catalonia, Spain) of the INMA (INfancia y Medio Ambiente) project. This project collects data of children and their families in seven cohorts across Spain, and its main objective is to analyze the association between early exposure to environmental factors and children’s physical and neuropsychological development and health [49]. For the present study, we used data of two cohorts due to data availability. The participants’ mothers were contacted during their first trimester of pregnancy in health centers or hospitals in the public health system. They were required to meet certain inclusion criteria, such as being over 16 years-old, having a single pregnancy, having the intention of giving birth in their referral hospital, not having communication problems, and not having followed an assisted reproduction program. The data was collected in multiple phases, including during pregnancy and birth, as well as at various ages for the child (14 months, 26 months, 4 years, 8 years and, 11 years of age). The ethical committees of the hospitals approved the project, and informed consent was obtained from all participants in each of the phases. The data used in this study was from the 11-year follow-up phase, where we collected data about bullying behavior. A total of 878 children and families were visited in this follow up phase, and after excluding individuals with missing bullying information (*n* = 3), HCC (Hair Cortisol Concentration) data (*n* = 212), or some covariates such as maternal educational level, or mainly pubertal status (*n* = 198), the final sample used in the analysis was 465. We explored the differences between participants included and excluded from the study regarding the main variables of the study (Appendix A). We observed differences only in fat mass percentage, specifically; those excluded from the study had lower fat percentage.

### Instruments

#### Anthropometric measures.

Weight and height were measured in preadolescents without shoes and in light clothing by trained staff and following a standard protocol. Body Mass Index (BMI) was calculated dividing weight in kilograms by height in meters squared (kg/m^2^). Additionally, trained staff assessed body fat percentage using bioimpedance and applying the equation proposed by Clasey et al. (2011) [50].

####  Hair cortisol.

Trained staff collected hair samples from the posterior vertex region of participants’ heads, following a guideline published by the Society of Hair Testing [51]. This area was chosen due to its consistent growth rate. The hair samples were placed in sealed plastic bags labeled with identification numbers and stored at room temperature until analysis. All analyses were conducted in the Clinical Chemistry Laboratory of the University of Linköping (Sweden). This hair segment reflects hair growth from the three months prior to sample collection [51]. Because hair typically grows at a rate of 1 cm per month, analyzing Hair Cortisol Concentration (HCC) in each cm provides month-to-month approximations of systemic cortisol levels. To account for potential external factors that might affect measurable levels of cortisol, such as frequent shampooing or hair bleaching products, participants were asked about these factors. Previous research showed that hair pigmentation may affect cortisol levels [52], however, since the majority of the sample population was Spanish, there was no need to control for this variable.

Hair cortisol was quantified using a competitive radioinmmunoassay (RIA), which involved meticulously processing the hair samples. The hair samples were first cut into small pieces and placed in 2mL QiaGenRB sample tubes with a 0.5 mm QuiGen stainless steel bead. The samples were then weighted on Sartorious MC 210p microscale and homogenized using a Retch Tissue Lyzer II (20 HZ). Next, the samples were frozen in liquid nitrogen for 2 min homogenization to produce fine hair powder. Subsequently, 1mL of methanol was added to each tube, and the samples were extracted overnight on a moving board. Afterwards, 0.8 mL of methanol supernatant was pipetted off and lyophilized using a Savant Speed Vas Plus SC210A. The samples were dissolved in radioimmunoassay buffer and analyzed. The primary antibody used was Rabitt Cortisol 3 Polyclonal Antibody (MyBiosource, San Diego, USA). The secondary antibody which was anti-rabbit IgG was Sac Cell AA-Sac 1 (InmmunoDiagnostic System Ltd, Bordon, England). It was required that hair samples weighed between 3 and 10 mg to maintain a total inter-assay coefficient of variation below 8% for hair extraction and measurement of cortisol by the radioimuunoasay. The method is fully described elsewhere [53].

#### **Bullying**.

Bullying was assessed using a shortened version of the Olweus Bully Victim Questionnaire (OBVQ) [54] at the 11 years’ old follow-up, and preadolescents were asked to respond based on their bullying experiences in the past two months. The OBVQ is a self-report instrument that has been widely used worldwide and has shown satisfactory psychometric properties [55]. For the current study, a short version was used, which includes a standardized definition of bullying and 16 questions. The first eight items assess various victimization behaviors (physical, verbal, social, sexual and cyberbullying) while the second eight pertain to physical, verbal, social, sexual, or cyber harassment to another student. The items were rated on a 5-point Likert scale (0 “it hasn’t happened to me in the past couple of months”, 1 “it happens once or a few times”, 2 “it happens 2 or 3 times a month (every month)”, 3 “it happens “every week” and 4 “it happens several times a week”). Those preadolescents who punctuated two or more in the Likert scale at least in one of the 8 questions of the first subscale were identified as victims, and those who scored equal or higher than two in at least one of the 8 questions that conformed to the second subscale, were identified as bullies. A third category (bully/victim) was created for those preadolescents who scored two or more in both subscales. A dichotomous variable was subsequently created, following criteria used by Solberg and Olweus (2003): (0) not involved or (1) frequently involved in bullying. The OBVQ showed adequate internal consistency in the present sample: α = 0.81 for the whole questionnaire, α = 0.82 for victim scale and α = 0.67 for bully scale [56].

#### **Covariates.**

The following covariates were included in the analysis: sex, cohort, maternal educational level (primary, secondary or university) and pubertal status. This last was assessed using Tanner stages [57, 58]. This scale differentiated five stages of development based on pubic hair and genital development in boys and pubic hair and breast development in girls. Parents were instructed to choose the drawing closest to their children stage of development.

### Data analysis

Using R software version 4.0.3, we conducted Structural Equation Modelling (SEM), particularly path analysis, as all variables used in the model were observed. This technique allows the examination of complex relationships within networks [59, 60]. Path analysis assumes linearity in the relationships between continuous variables and Gaussian error terms. In the first step, we assessed the symmetry of each relevant variable, transforming data, when appropriate. This transformation was performed to ensure the linearity of relationships and hence the suitability of the global estimation method.

**Fig. 1:**
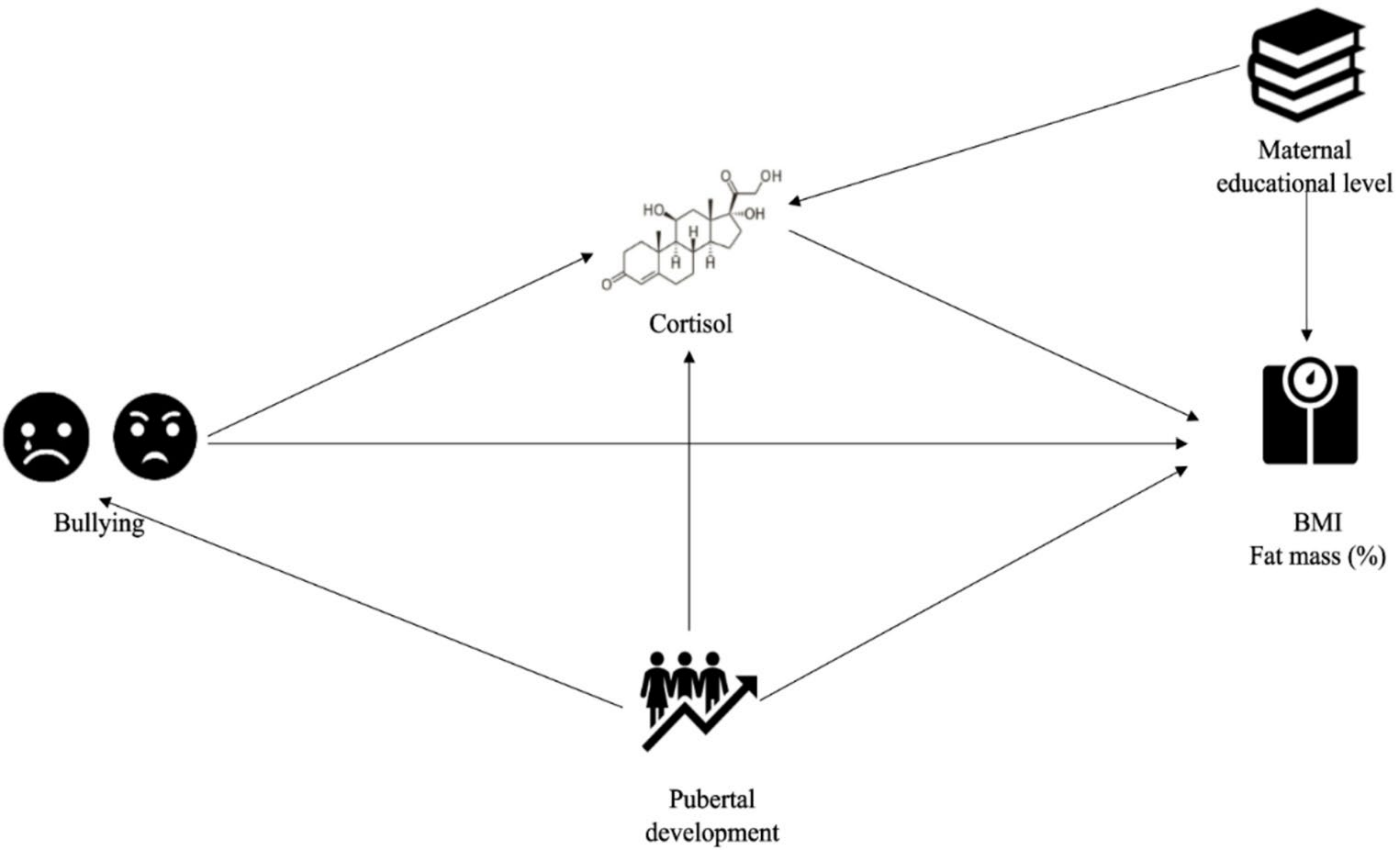
Metamodel summarizing hypothesized relationship between bullying and weight status mediated by cortisol

Initially, we examined the descriptive statistics, and we conducted a bivariate analysis. After constructing the metamodel (Fig. 1), which was based on a priori theoretical knowledge, we conducted an exploratory data analysis to verify that the assumptions required were met. We then obtained global estimates using the maximum likelihood estimation method. Model fit was assessed using a Chi-square test, which compared the tested model with a saturated model. Apart from the chi-square test, the Root Mean Square Error of Approximation (RMSEA) was used [61]. The final models were accepted only when the following conditions were met: Chi-square test *p-*value > 0.05, a Comparative Fit Index (CFI) > 0.95 and, RMSEA < 0.05.

We conducted separate models for each outcome (i.e., BMI and body fat percentage). In total, we carried out a total of twelve models, one per each role that preadolescents could take in bullying (victim, bully or bully/victim) and separated by sex.

## Metamodel description

The primary objective of the study was to explore whether preadolescents’ hair cortisol concentration (HCC) mediated the relationship between involvement in bullying and weight status (BMI and body fat percentage). Additionally, we sought to investigate the role of maternal education level and pubertal status in relation not only to bullying behavior but also to cortisol levels and weight status.

Based on existing literature, we constructed the metamodel (Fig. 1). Previous evidence has identified an association between childhood victimization and later development of overweight or obesity in the adulthood [42, 43], but only one study analyzes stress as possible mediator in the association between school environment and weight status [26]. Regarding pubertal status, previous literature posits that early pubertal timing is related to risky behaviors and higher BMI during adolescence, particularly during adolescence [62].

Furthermore, as sex has been shown to influence cortisol levels [63, 64], bullying behavior [65] and weight-related variables [66], we decided to conduct separated model for boys and girls.

## Results

### Sample description

We conducted an initial analysis to assess differences between the cohorts and we found that participants from Sabadell were older, and were more pubertally developed (higher score in Tanner stages). However, participants from Gipuzkoa presented a higher fat mass percentage and their mothers presented a higher educational level.

The study sample consisted of 465 preadolescents (58.3% girls and 41.7% boys), with a mean age of 11 years (*M =* 10.9; *SD =* 0.42) from Gipuzkoa (*n =* 336) and Sabadell (*n =* 129). In terms of bullying prevalence, 8.6% of the participants were involved as victims, 1.7% as bullies and 2.4% as bully/victims. Regarding weight status, following WHO BMI z-score classification, 1.1% (*n* = 5) of the participants had thinness, 62.7% (*n* = 292) were normal, 24.9% (*n* = 116) were overweight and 11.4% (*n* = 53) had obesity. The fat percentage of preadolescents was found to be higher than 25% (*M* = 27.3; *SD* = 7.54).

### Sex differences

When analyzing sex differences, we observed that girls had a higher percentage of body fat percentage and were more advanced in pubertal development, as indicated by higher scores in Tanner stages. Furthermore, a greater number of boys were frequently involved as bullies/victims (Table 1).

**Table 1:** Sex differences

Variable		Female (*n* = 271)		Male (*n* = 194)		Statistic; *p*-value
		M (SD)	*F* (%)	M (SD)	*F* (%)	
Age		10.89 (0.43)		10.92 (0.39)		*t* (463) = –0.656; *p* = 0.512
Cortisol ln		2.23 (0.48)		2.26 (0.45)		*t* (463) = –0.5843; *p* = 0.280
Maternal educational level	Primary		37 (13.7)		23(11.9)	Chi (1) = 0.328; *p* = 0.849
	Secondary		102 (37.6)		75(38.7)	
	University		132(48.7)		96 (49.5)	
BMI		19.07 (3.07)		19.03 (3.49)		*t* (463) = 0.253; *p* = 0.400
Fat mass (%)		28.27 (7.37)		26.13 (7.61)		***t*** **(463) = 3.032;** ***p*** ** = 0.001**
Tanner	1	53(19.6)		108 (55.7)		**Chi (4) = 81.435;** ***p*** **< 0.001**
	2	124(45.8)		71 (36.6)		
	3	55 (20.3)		12 (6.2)		
	4	28 (10.3)		3 (1.5)		
	5	11 (4.1)		0(0)		
Victim	Not involved		248 (91.5)		177 (91.2)	Chi (1) = 0.011; *p* = 0.917
	Involved		23 (8.5)		17 (8.8)	
Bully	Not involved		266 (98.2)		191 (98.5)	Chi (1) = 0.060; *p* = 0.807
	Involved		5 (1.8)		3 (1.5)	
Bully victim	Not involved		268 (98.9)		186 (95.9)	**Chi (1) = 4.455;** ***p*** **= 0.035**
	Involved		3 (1.1)		8 (4.1)	

## Path analysis

### Females

**Fig. 2:**
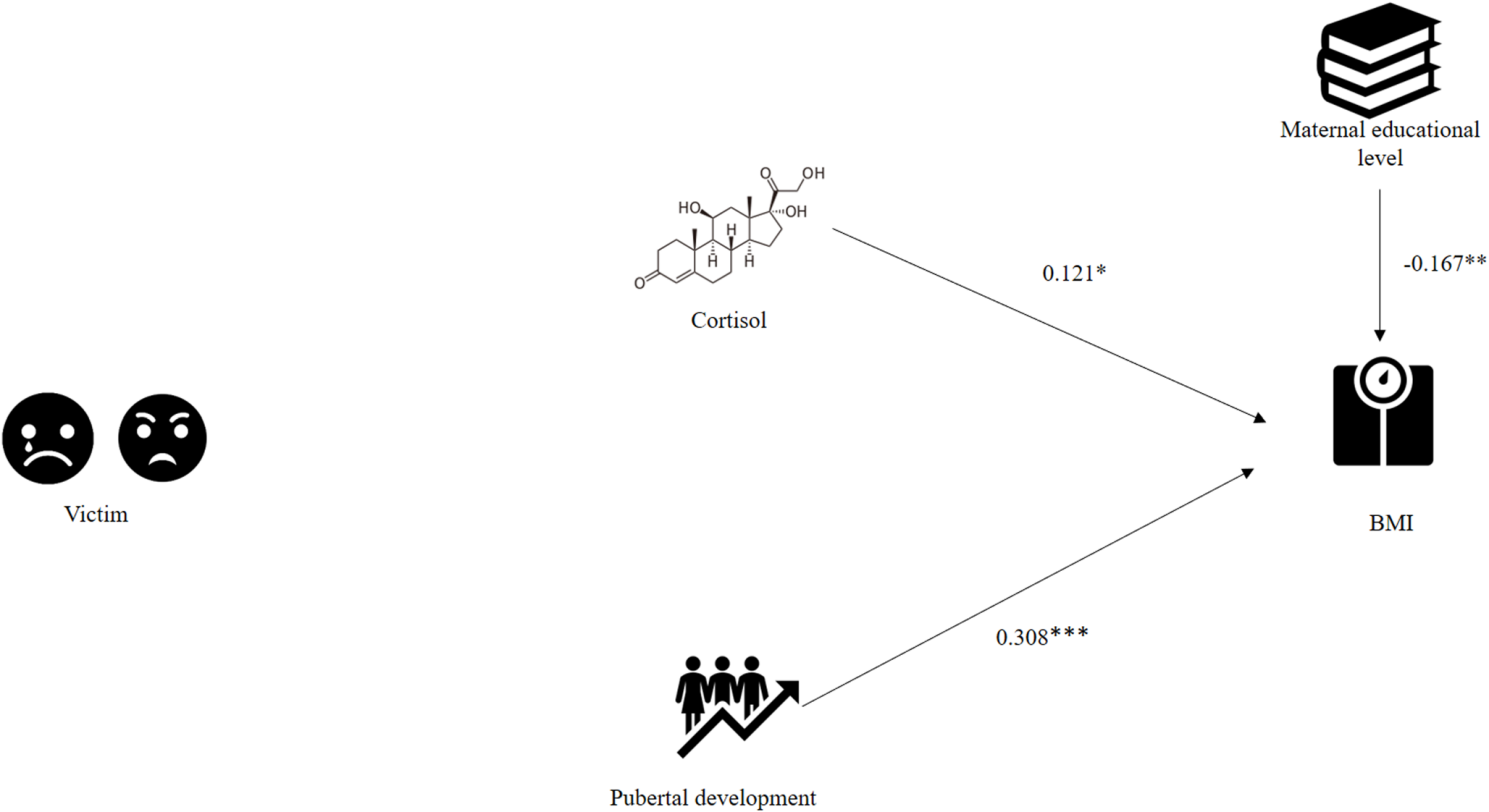
Final model for female victims using Body Mass Index as an outcome, where standardized coefficients estimates are shown. •*p*<0.10; *p<0.05; **p<0.01; ***p<0.001

For girls, valid models were obtained only for the role of victim (Fig.2, Table 2). The results of the model testing the mediating effect of cortisol in the association between victim role and BMI showed a good fit between the model and data (*X*^2^ (1) = 0.00; *p* = 0.982; CFI = 1.00; RMSEA = 0.000; *p* = 0.987). The results indicate that higher HHC were related to higher BMI (*b* = 0.086; *p =* 0.047). Moreover, we observed that being more pubertally developed was associated to higher BMI (*b* = 0.102; *p <* 0.001). Lastly, it was noted that those girls whose mothers had lower educational levels had higher BMI *(b*=-0.081; *p* = 0.005).

**Table 2:** Final model for female victims using body mass index as an outcome

Variable	Predictor	Estimate	St.Err	z-value	*p*-value
BMI					
	Cortisol	0.086	0.043	1.988	**0.047**
	Victim	-0.003	0.035	-0.087	0.931
	Maternal study level	-0.081	0.029	-2.785	**0.005**
	Pubertal status	0.102	0.020	4.992	**< 0.001**
Cortisol					
	Victim	-0.024	0.069	-0.347	0.728
	Pubertal status	0.002	0.035	0.064	0.949
	Maternal study level	0.030	0.038	0.803	0.422
	Pubertal status	-0.039	0.105	-0.371	0.711

**Fig. 3:**
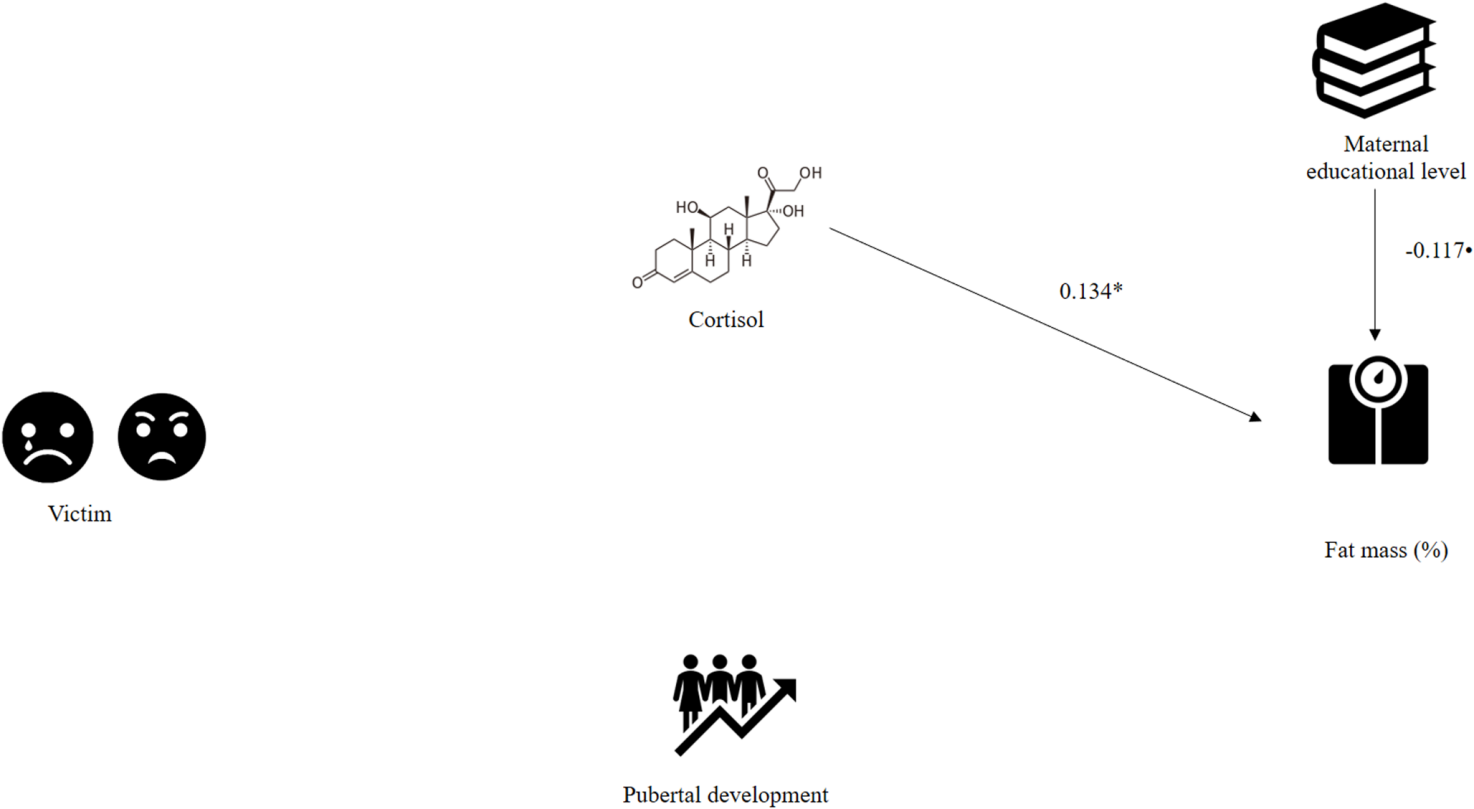
Final model for female victims using fat percentage as an outcome, where standardized coefficients estimates are shown. •*p*<0.10; *p<0.05; **p<0.01; ***p<0.001

On the other hand, the results from the model testing role of cortisol in the association between victim role and fat percentage of fat (Fig.3, Table 3) mass showed a good fit between the model and data (*X*^2^ (1) = 0.00; *p* = 0.982; CFI = 1.00; RMSEA = 0.000; *p* = 0.987). The findings showed that as well as happened with BMI,, similar to BMI, higher hair cortisol levels were related to associated with a higher fat mass percentage of fat mass (*b* = 2.049; *p =* 0.033). Additionally, we observed that those girls whose mothers had lower study levels had higher fat mass percentage, although this association was not statistically significant *(b* = 1.215; *p* = 0.07).

**Table 3:** Final model for female victims using fat percentage as an outcome

Variable	Predictor	Estimate	Std.Err	z-value	*p*-value
Fat mass					
	Cortisol	2.049	0.959	2.136	**0.033**
	Victim	0.512	0.815	0.629	0.529
	Maternal educational level	-1.215	0.686	-1.772	0.07*
	Pubertal status	0.447	0.455	0.983	0.326
Cortisol					
	Victim	-0.024	0.069	-0.347	0.728
	Pubertal status	0.002	0.035	0.064	0.949
	Maternal educational level	0.030	0.038	0.803	0.422
	Pubertal status	-0.039	0.105	-0.371	0.711

### Males

**Fig. 4:**
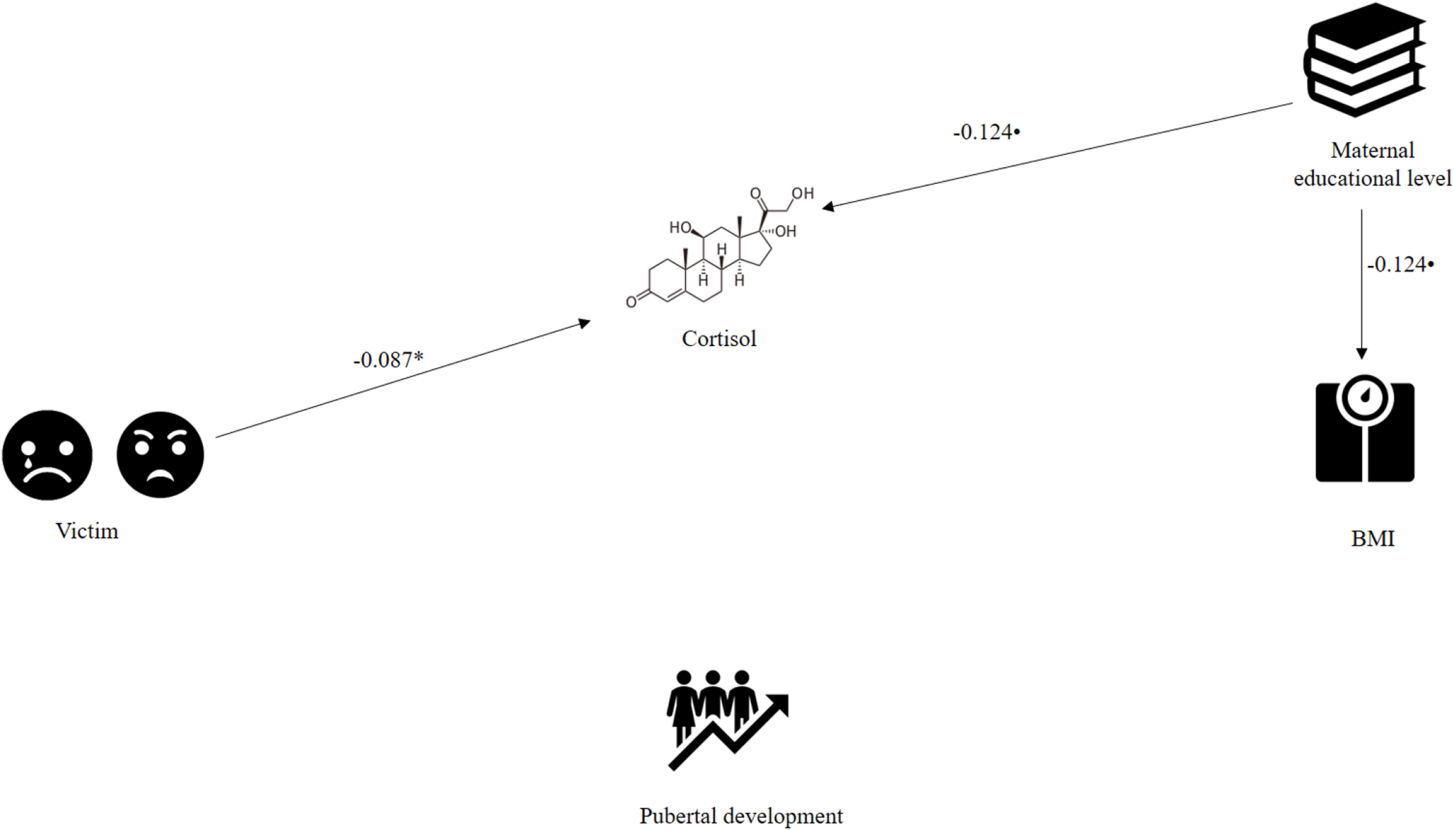
Final model for male victims using BMI as an outcome, where standardized coefficients estimates are shown. •*p*<0.10; *p<0.05; **p<0.01; ***p<0.001

For boys, valid models were obtained only for the victim role (Fig.4, Table 4). The results of the model testing role of cortisol in the association between victim role and BMI showed a good fit between the model and data (*X*^2^ (1) = 0.326; *p* = 0.568; CFI = 1.00; RMSEA = 0.000; *p* = 0.652). Results indicate that lower maternal study level was related with higher BMI (*b=*-0.07; *p =* 0.06) and to higher cortisol levels (*b=*-0.082; *p =* 0.08). Finally, being involved as a victim was related to lower cortisol levels (*b=*-0.039; *p =* 0.05).

**Table 4:** Final model for male victims using body mass index as an outcome

Variable	Predictor	Estimate	St.Err	z-value	*p*-value
BMI					
	Cortisol	-0.031	0.069	-0.451	0.652
	Victim	0.035	0.041	0.849	0.396
	Maternal study level	-0.070	0.037	-1.881	**0.063**
	Pubertal status	0.051	0.046	1.118	0.264
Cortisol					
	Victim	-0.039	0.020	-1.932	**0.053**
	Pubertal status	-0.025	0.069	-0.355	0.723
	Maternal study level	-0.082	0.048	-1.720	**0.085**
	Pubertal status	0.192	0.159	0.985	0.324

**Fig. 5:**
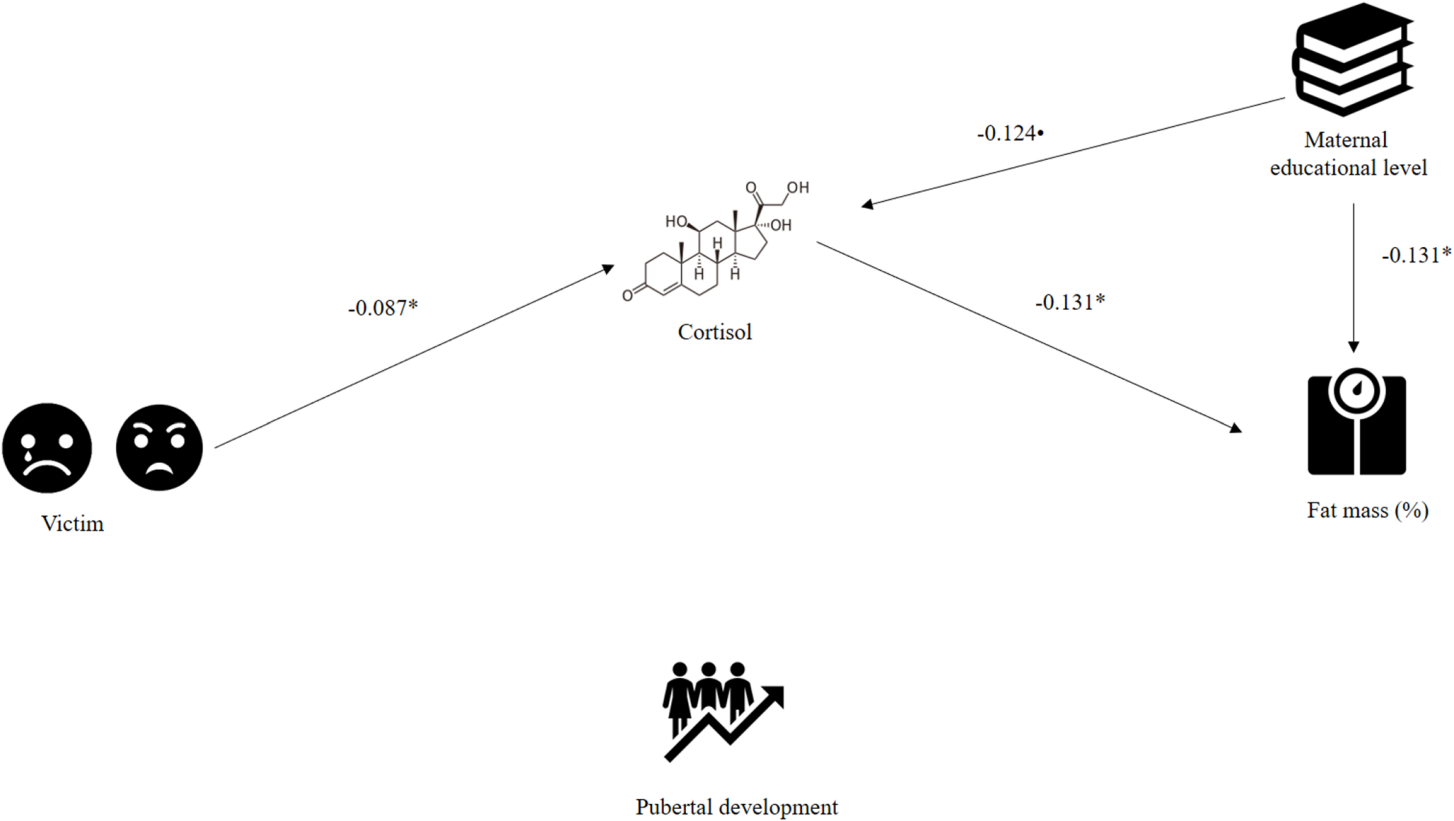
Final model for male victims using fat percentage as an outcome, where standardized coefficients estimates are shown. •*p*<0.10; *p<0.05; **p<0.01; ***p<0.001

The results from the model testing the role of cortisol in the association between victim role and percentage of fat mass (Fig.5, Table 5) indicated a good fit between the model and the data (*X*^2^ (1) = 0.326; *p* = 0.982; CFI = 1.00; RMSEA = 0.000; *p* = 0.987). These findings support our hypothesis was confirmed, as being involved as a victim associated with cortisol levels and, which in addition, cortisol levels were related to fat mass. Specifically, the results showed that being involved as a victim was linked to lower cortisol levels (*b=*-0.087; *p* = 0.05) and that lower hair cortisol concentration (HCC) was related to higher fat mass levels (*b*=-0.131; *p =* 0.05). While the direct effect of victimization on the percentage of fat mass was not statistically significant, the indirect effect mediated by cortisol was significant and positive (*b =* 0.011; *p <* 0.05). Finally, the model also showed that maternal educational level influence not only cortisol levels *(b*=-0.082; *p* = 0.08) but also preadolescents’ body mass percentage (*b* = 1.44; *p* = 0.05).

**Table 5:** Final model for male victims using fat mass as an outcome

Variable	Predictor	Estimate	St.Err	z-value	*p*-value
Fat mass					
	Cortisol	-2.202	1.144	-1.925	**0.054**
	Victim	0.390	0.871	0.448	0.654
	Maternal study level	-1.449	0.760	-1.907	**0.057**
	Pubertal status	-0.462	0.884	-0.523	0.601
Cortisol					
	Victim	-0.039	0.020	-1.932	**0.053**
	Pubertal status	-0.025		-0.355	0.723
	Maternal study level	-0.082	0.048	-1.720	**0.085**
	Pubertal status	0.192	0.195	0.985	0.324

## Discussion

The aim of this study was to investigate the mediating role of cortisol in the relationship between bullying and weight status. Specifically, we sought to analyze gender differences and examine how bullying might influence weight and body fat distribution. To the best of our knowledge, this is the first research exploring this relationship, as only one previous study has examined the role of perceived stress as a mediator between the school environment and obesity.

When examining gender differences, our results indicated that girls exhibited a higher percentage of fat mass, as well as greater pubertal development, as assessed by Tanner stages. Concerning body composition during adolescence, previous studies showed that girls tend to have a higher percentage of fat mass compared to boys of similar ages [67].

The main hypothesis of the study was partially confirmed, as a mediating effect of cortisol between bullying involvement and fat percentage was identified in boys, but only for the role of victimization. This means that, while no direct effect was found between bullying and fat mass, an indirect effect was present. In other words, being a victim of bullying is associated with higher fat mass via cortisol levels. This study is the first exploring the mediating role of stress in the association between school bullying and weight status, using cortisol levels as a biomarker of stress. Previous research indicated that the relationship between individual-level school context and overweight was mitigated by stress but this association was only observed in girls [26]. In comparison to our study focused on adolescents of 16 years and explored the association between school context (not only bullying) and BMI, and used perceived stress as mediating variable. Nevertheless, both cases showed sex specific response in the explored association. While in our study the hypothesis was only observed in boys, studies involving girls suggest that cortisol levels are also linked to their weight status. Although bullying may not have influenced this relationship, it is possible that other stressors are affecting this association.

This finding in our study highlights an important distinction between boys and girls in relation to bullying. In boys, being a victim of bullying seems to be linked to changes in the physiological stress response, specifically measured by HCC. However, this association has not been observed in girls. The differences in stress responses may be explained by traditional theories: boys tend to exhibit a ‘fight or flight’ response, leading to an immediate physiological reaction with alterations in cortisol levels due to confrontational or avoidance behaviors. In contrast, girls are more likely to respond with a ‘tend and befriend’ approach, which may involve emotional or social coping strategies that have less direct impact on cortisol levels [68]. Additionally, these differences could also be influenced by hormonal, social, and cultural factors that shape how each gender perceives and responds to bullying.

Despite its innovative contributions, this study is not without limitations. One primary limitation is the sample size and its characteristics, particularly the lack of age variability and so, pubertal status. This limitation also affected the balance of bullying-related variables, with some categories being overrepresented. Moreover, the excellent fit indices observed (CFI = 1.00; RMSEA = 0.000) should be interpreted with caution, as they may reflect the small sample sizes rather than true model fit. Thus, the generalization of the results may be considered cautiously. In addition, participants included in the study showed higher fat percentage in comparison to those not included in the study. Furthermore, while the role of cortisol has been analyzed, other potential biological markers that could provide further insights into mechanisms at play, such as inflammatory factors or neurotransmitters activated in response to stress. Finally, the cross-sectional and exploratory nature of the study prevents drawing causal conclusions, and results should be considered as preliminary evidence.

Nevertheless, the study have several strengths. To the best of our knowledge, this is the first study investigating the mediating role of cortisol in the association between bullying and weight status. This opens avenues for further research into the potentially harmful effects of bullying as a significant stressor for adolescents and its impact on weight status. Additionally, this study has explored the different roles of bullying, as well as BMI and fat mass, in understanding weight status in adolescents. These findings highlight the importance of considering mediating variables when analyzing complex relationships between different factors.

## Conclusions

Most of the previous research focus demonstrated that overweight children are bullied by their peers. However, only one previous study explored how being involved in bullying affects weight status via stress response. To the best of our knowledge the present study is the first analyzing this mediation using a biomarker of the physiological stress response. While we found evidence of mediating effect of stress responses (HCC) in the relationship between bullying victimization and weight status, this association was only confirmed for boys. Importantly, given the methodological limitations of our study—including small sample sizes and cross-sectional design—these findings should be interpreted as exploratory. They provide a preliminary hypothesis that future studies with larger, more diverse samples and longitudinal designs can test. Nonetheless, our study highlights the potential biological embedding of social stressors such as bullying and underscores the importance of investigating physiological stress mechanisms in the context of weight-related outcomes.

## Data Availability

No datasets were generated or analysed during the current study.

## Appendix

**Table 6:** Differences in the main study variables between included and non-included participants

Variable		Included (*n* = 465)			Excluded (*n* = 413)		Statistic
		M (SD)	F (%)		M (SD)	F (%)	
BMI z score		0.61 (1.14)			0.72 (1.21)		*t* (876) = 1.366; *p* = 0.087
BMI		19.05 (3.25)			19.40 (3.51)		*t* (876) = 843.63; *p* = 0.063
Fat mass (%)		27.38 (7.54)			25.44 (7.68)		*t* (876)=-3.768; *p* < 0.001
Victim	Not involved		426 (91.6)			367 (89.5)	*Ch*i (1) = 1.132; *p* = 0.287
	Involved		39 (8.4)			43 (10.5)	
Bully	Not involved		457 (98.3)			405 (98.8)	*Chi* (1) = 0.375; *p* = 0.541
	Involved		8 (1.7)			5 (1.2)	
Bully_victim	Not involved		454 (97.6)			406 (99)	*Chi* (1) = 2.498; *p* = 0,114
	Involved		11 (2.4)			4 (1)	
